# Development and validation of an interpretable clinical score for early identification of acute kidney injury at the emergency department

**DOI:** 10.1038/s41598-022-11129-4

**Published:** 2022-05-02

**Authors:** Yukai Ang, Siqi Li, Marcus Eng Hock Ong, Feng Xie, Su Hooi Teo, Lina Choong, Riece Koniman, Bibhas Chakraborty, Andrew Fu Wah Ho, Nan Liu

**Affiliations:** 1 Duke-NUS Medical School, National University of Singapore, Singapore, Singapore; 2 Department of Emergency Medicine, Singapore General Hospital, Singapore, Singapore; 3 Department of Renal Medicine, Singapore General Hospital, Singapore, Singapore; 4 Department of Biostatistics & Bioinformatics, Duke University, Durham, NC USA; 5 Department of Statistics and Data Science, National University of Singapore, Singapore, Singapore; 6 Health Service Research Centre, Singapore Health Services, Singapore, Singapore; 7 SingHealth AI Health Program, Singapore Health Services, Singapore, Singapore; 8 Institute of Data Science, National University of Singapore, Singapore, Singapore; 9 Programme in Health Services and Systems Research, Duke-NUS Medical School, 8 College Road, Singapore, 169857 Singapore

**Keywords:** Health care, Health services

## Abstract

Acute kidney injury (AKI) in hospitalised patients is a common syndrome associated with poorer patient outcomes. Clinical risk scores can be used for the early identification of patients at risk of AKI. We conducted a retrospective study using electronic health records of Singapore General Hospital emergency department patients who were admitted from 2008 to 2016. The primary outcome was inpatient AKI of any stage within 7 days of admission based on the Kidney Disease Improving Global Outcome (KDIGO) 2012 guidelines. A machine learning-based framework AutoScore was used to generate clinical scores from the study sample which was randomly divided into training, validation and testing cohorts. Model performance was evaluated using area under the curve (AUC). Among the 119,468 admissions, 10,693 (9.0%) developed AKI. 8491 were stage 1 (79.4%), 906 stage 2 (8.5%) and 1296 stage 3 (12.1%). The AKI Risk Score (AKI-RiSc) was a summation of the integer scores of 6 variables: serum creatinine, serum bicarbonate, pulse, systolic blood pressure, diastolic blood pressure, and age. AUC of AKI-RiSc was 0.730 (95% CI 0.714–0.747), outperforming an existing AKI Prediction Score model which achieved AUC of 0.665 (95% CI 0.646–0.679) on the testing cohort. At a cut-off of 4 points, AKI-RiSc had a sensitivity of 82.6% and specificity of 46.7%. AKI-RiSc is a simple clinical score that can be easily implemented on the ground for early identification of AKI and potentially be applied in international settings.

## Introduction

Acute kidney injury (AKI) is a common clinical syndrome affecting 10% to 20% of hospitalized patients worldwide^1^. It is independently associated with increased risk of inpatient mortality^2^, significant morbidity upon discharge^3^, and increased healthcare costs as well as length of hospital stay^4^. AKI is also often clinically silent and may not be promptly recognised by the attending physician^5^. Established kidney injury is often difficult to treat^6^, hence there is a need for early detection to initiate treatment promptly.

AKI is typically diagnosed by the magnitude of serum creatinine (SCr) rise and is usually based on well accepted criterion such as the Kidney Disease Improving Global Outcome AKI (2012) guidelines. Early warning systems have been developed to flag patients with pathological rises in SCr, which can then be paired with intervention care bundles that can prevent the progression of AKI^7^. However, increase in SCr levels can lag kidney injury by up to 48 h^8^. Physicians, therefore, are only reacting to damage that has already been done.

AKI prediction models are potential solutions to this problem. In recent years, there has been increasing interest in the development of machine learning (ML) models, which can accurately predict AKI development in patients before any rise in SCr^9–11^. Physicians will then be able to intervene earlier and halt the progression of the renal insult^12^. However, these ML models have yet to be widely utilized in clinical practice due to their complexity which may be difficult to implement in existing hospital information technology (IT) systems^11^. Furthermore, many ML models derive their predictions via black box approaches, which are not always explainable to humans in a rational way. Hence, clinicians may not be as inclined to apply them in clinical practice^13^. Point-based prediction scores offer a simple and interpretable solution to these ML models. However, most existing point-based AKI risk scores were designed for specific patient populations in the Intensive Care Unit^14,15^, post-operative period^16^, and undergoing procedures involving contrast^16–18^, when the majority of AKI occurs in the general ward setting^19^.

The primary aim of this study was to create a simple point-based clinical AKI Risk Score (AKI-RiSc) for the general patient population using a systematic, machine learning-based scoring framework—AutoScore^20^. The AKI-RiSc was designed to assess a patient’s 7 day inpatient risk of AKI development in the setting of the emergency department (ED). It is envisioned that AKI-RiSc can function as a convenient and informative adjunct in allowing clinicians to assess a patient’s risk of inpatient AKI and institute any necessary management steps more accurately.

## Methods

### Study design and setting

We conducted a retrospective, single-centre study in Singapore General Hospital (SGH) to derive AKI-RiSc using electronic health record (EHR) of the patients in the ED and inpatient wards. SGH is a tertiary hospital that receives over 120,000 ED visits and 36,000 admissions annually. A waiver of consent for EHR data collection was granted, and the study protocol was approved by Singapore Health Services’ Centralised Institutional Review Board. All research has been performed in accordance with the Declaration of Helsinki.

### Study sample

All adult patients (> 18 years old) visiting the ED between 1 January 2008 and 31 December 2016 and who were subsequently admitted to medical and surgical wards were studied^21^. Patients were excluded if they met any of the following criteria: (1) patients with no records of SCr, (2) patients with AKI on presentation at the ED, defined based on KDIGO change of SCr from median annualised SCr baseline, (3) patients with pre-existing advanced chronic kidney disease (CKD) based on KDIGO guidelines (SCr ≥ 353.6 µmol/L on admission) and (4) patients with no information of comorbidities. Patients were then followed up to 7 days post-admission to determine if they developed AKI or not.

### AKI definition and outcomes

The primary outcome of this study was the development of inpatient AKI of any stage within 7 days of admission from the ED. We used the National Health Service (NHS) automated AKI algorithm based on KDIGO guidelines to define AKI^22^. The algorithm had been applied in clinical practice and was chosen because of its ability to account for patients with and without prior baseline SCr information^23^. A patient was determined to have AKI if any of the three criteria were met: (1) Increase in SCr to ≥ 1.5 times of the median of all SCr readings 8 to 365 days ago, (2) increase in SCr to ≥ 1.5 times of the lowest SCr reading in the past 7 days, (3) increase in SCr by ≥ 26.5 µmol/L within 48 h from the lowest SCr reading. Other outcomes that were examined in this study were patients who developed at least Stage 2 AKI and patients who developed Stage 3 AKI.

### Data collection and candidate variables

We extracted the data from the hospital’s EHR—SingHealth Electronic Health Intelligence System. The data was de-identified, and the death records were obtained from the national death registry and matched to specific patients in the EHR. As the AKI-RiSc was designed to be applied in the ED setting, we only selected variables that were exclusively available and reliably obtained at the ED. The final 33 candidate variables were selected based on literature review and expert opinion from clinicians, which consisted of patient demographics, vital signs, biochemical results, comorbidities, medical interventions, and visits to hospital—as seen in Table 1. Comorbidities were obtained from the hospital diagnosis and discharge records in the past five years before each patient’s current ED visits. They were recorded as the International Classification of Diseases (ICD) 9/10 codes^24^.

**Table 1:** Comparison of patient characteristics between those who developed AKI 7 days after admission and those who did not at the emergency department.

Variable	No AKI in 7 days (n = 108,775)	AKI in 7 days (n = 10,693)	P-value
Age, year, mean (SD)	65.77 (17.23)	70.17 (14.21)	< 0.001
Female, n (%)	53,692 (49.4)	5277 (49.4)	0.991
Intubation (mean (SD))	40 (0.0)	16 (0.1)	< 0.001
Resuscitation (mean (SD))	2986 (2.7)	601 (5.6)	< 0.001
Pulse, mean (SD)	84.31 (17.62)	88.28 (19.67)	< 0.001
Respiration, mean (SD)	17.89 (1.76)	18.29 (2.45)	< 0.001
SpO_2_, mean (SD)	97.97 (2.94)	97.74 (3.49)	< 0.001
Diastolic blood pressure, mean (SD)	69.83 (13.13)	70.04 (14.93)	0.113
Systolic blood pressure, mean (SD)	132.03 (23.97)	135.23 (27.27)	< 0.001
Bicarbonate, mmol/L, mean (SD)	22.47 (3.94)	21.29 (4.51)	< 0.001
Creatinine, μmol/L**,** mean (SD)	104.67 (59.14)	159.92 (87.53)	< 0.001
Potassium, mmol/L, mean (SD)	4.15 (0.80)	4.27 (0.86)	< 0.001
Sodium, mmol/L, mean (SD)	133.45 (6.13)	133.63 (6.58)	0.006
Myocardial infarction, n (%)	6496 (6.0)	1879 (17.6)	< 0.001
Congestive heart failure, n (%)	15,178 (14.0)	3355 (31.4)	< 0.001
Peripheral vascular disease, n (%)	5568 (5.1)	1267 (11.8)	< 0.001
Stroke, n (%)	14,665 (13.5)	2000 (18.7)	< 0.001
Dementia, n (%)	3742 (3.4)	427 (4.0)	0.003
Pulmonary disease, n (%)	11,607 (10.7)	1525 (14.3)	< 0.001
Rheumatic disease, n (%)	1464 (1.3)	205 (1.9)	< 0.001
Peptic ulcer disease, n (%)	4637 (4.3)	732 (6.8)	< 0.001
Mild liver disease, n (%)	5706 (5.2)	746 (7.0)	< 0.001
Diabetes mellitus, n (%)	18,233 (16.8)	2045 (19.1)	< 0.001
Diabetes complications, n (%)	26,623 (24.5)	3956 (37.0)	< 0.001
Severe liver disease, n (%)	2192 (2.0)	359 (3.4)	< 0.001
Paralysis, n (%)	6380 (5.9)	862 (8.1)	< 0.001
Renal disease, n (%)	18,386 (16.9)	4844 (45.3)	< 0.001
Cancer, n (%)	11,408 (10.5)	1279 (12.0)	< 0.001
Metastatic disease, n (%)	9566 (8.8)	1053 (9.8)	< 0.001
No. of visits in last year, mean (SD)	0.85 (1.94)	1.20 (2.11)	< 0.001
No. of ICU admits last year, mean (SD)	0.02 (0.23)	0.05 (0.37)	< 0.001
No. of surgery last year, mean (SD)	0.15 (0.59)	0.28 (0.88)	< 0.001
No. of HD admits last year, mean (SD)	0.08 (0.44)	0.14 (0.61)	< 0.001
Inpatient mortality, n (%)	3887 (3.6)	2327 (21.8)	< 0.001

*AKI* acute kidney injury, *SpO*_*2 *_oxygen saturation, *HD* high dependency unit.

### Statistical analyses and predictive modelling

**Figure 1:**
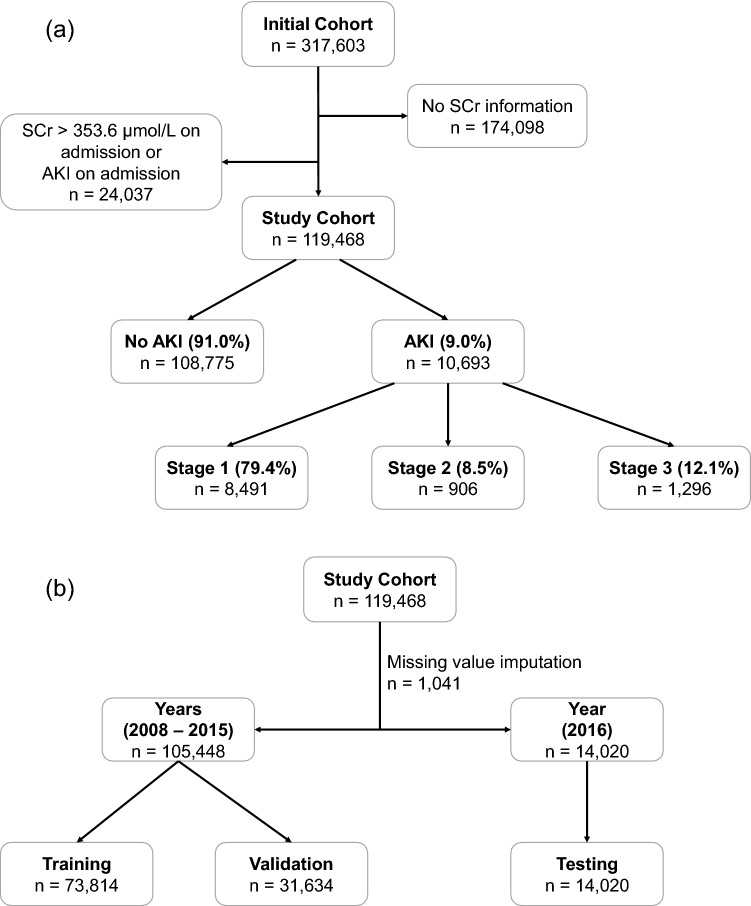
Flowcharts of (**a**) patient selection, and (**b**) splitting of study cohort into training, validation and testing cohorts.

Variables were included for the analysis only if more than 80% of them were available in the study cohort. These missing values were replaced by the mean if the variable was continuous or by the most frequent category if the variable was categorical. Chi-square for categorical variables and *t-*tests for continuous variables were used where appropriate to compare between the patients who developed AKI and those who did not. Univariable analysis was also used to determine the odds ratios of risk factors between the two groups. Figure 1a depicts the flow of patient selection. Patients admitted between 2008 and 2015 were randomly divided into training and validation cohorts, at a 70% and 30% ratio, respectively (Fig. 1b). Patients admitted in 2016 were used as the testing cohort to evaluate the final score.

AKI-RiSc was developed using AutoScore^20^, a machine learning-based algorithm for interpretable clinical score generation. AutoScore facilitates the easy and transparent development of interpretable clinical score models for a pre-defined clinical outcome. It uses a combination of machine learning, logistic regression, and user-defined parameter fine-tuning. By implementing machine learning-based variable ranking and model selection, AutoScore can effectively handle the issue of variable multicollinearity. Figure S2 illustrates the process of score derivation and validation in the AutoScore framework.

The training cohort was first used to generate the preliminary AKI-RiSc models using the AutoScore R package. The validation cohort was then used to evaluate the performance of various candidate AKI-RiSc models and allow for parameter fine-tuning. After selecting the final AKI-RiSc model, we evaluated its performance using the testing cohort, and bootstrapped samples were applied to calculate 95% confidence intervals (CIs). The primary outcome measure of inpatient AKI development was used for model derivation and model testing. The predictive power of AKI-RiSc was assessed using the area under the curve (AUC) in the receiver operating characteristic (ROC) analysis. Sensitivity, specificity, positive predictive value (PPV), and negative predictive value (NPV) based on various AKI-RiSc cut-offs were also calculated. Additionally, we compared AKI-RiSc with an existing AKI clinical score, the AKI Prediction Score (APS)^25^, by evaluating its performance using the same testing cohort with the same primary outcome measure.

## Results

The initial cohort comprised 317,603 unique visits to the ED that were subsequently admitted. After excluding admissions with no available SCr (n = 174,098), and those with SCr > 353.6 µmol/L or AKI on admission (n = 24,037), we arrived at a final cohort of 119,468 unique entries. Among this cohort, 10,693 (9.0%) developed AKI of any stage within 7 days of admission, of which 8491 were stage 1 (79.4%), 906 stage 2 (8.5%), and 1296 stage 3 (12.1%) (Fig. 1a). The overall in-hospital mortality rate of patients admitted with AKI was 21.8%, as opposed to 3.6% in non-AKI counterparts. While most AKI patients were stage 1, the number of patients in stage 3 was higher than stage 2, which was also observed in another recent hospital-wide AKI study^26^.

Patients who developed AKI were older, had higher baseline SCr, higher admission SCr, and had more comorbidities associated with them (Table 1). Univariable analysis (Table S1) identified tachycardia, tachypnoea, hypotension, and hypertension as the most significant risk factors among the vital signs. Higher SCr levels on admission at the ED also increased the risk of inpatient AKI. Among the other lab values, low serum bicarbonate was the most significant risk factor for developing AKI. Extremes of serum sodium and potassium values also conferred an increased risk of AKI. All comorbidities were significantly associated with the development of AKI, but patients with renal disease had the greatest risk (odds ratio [OR] 4.07, 95% CI 3.91–4.24). Previous surgeries, admissions to the hospital, high dependency unit, and intensive care unit (ICU) were also significant risk factors.

**Figure 2:**
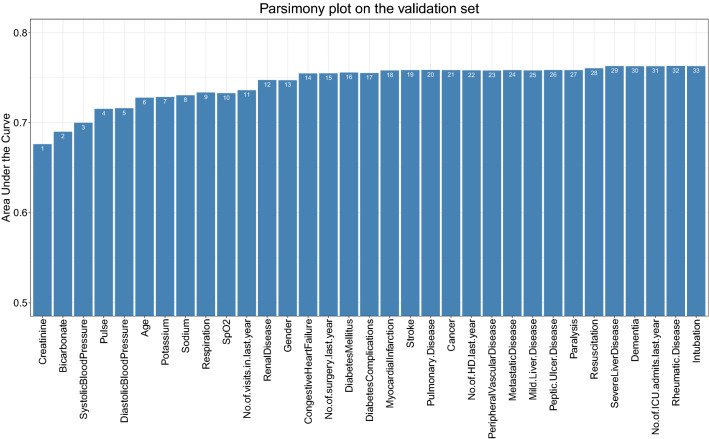
Parsimony plot of variables and predictive performance of model on the validation set. *SpO*_*2*_ oxygen saturation.

AKI-RiSc was developed in a stepwise manner with the AutoScore framework. After ranking the essential AKI prediction variables, a parsimony plot (Fig. 2) was created to visualise model selection. The variables shown in Fig. 2 were ranked in order of importance of AKI prediction based on the initial random forest selection, with SCr being the most important and intubation being the least. Figure 2 also plots the model performance (AUC) against model complexity (number of variables). The most significant increase in model performance was observed from the first to the sixth variable, with marginal gains in performance fort every other variable added thereafter.

We selected the six most important variables in the parsimony plot (SCr, serum bicarbonate, pulse, systolic blood pressure, diastolic blood pressure, and age) to build the AKI-RiSc. Each variable was allocated an integer score based on its importance. The final score was a summation of all the variable’s assigned scores, ranging from 0 to 15 (Table 2). Risk of inpatient AKI development was positively correlated with the higher AKI-RiSc scores. Higher SCr levels contributed most significantly with greater final scores, with SCr values ≥ 250 μmol/L being allocated the highest score of 7. Tachycardia (≥ 120 beats per minute) was also given a higher score of 3. Other variables associated with higher scores were lower bicarbonate levels of < 20 mmol/L, systolic blood pressure of ≥ 150 mmHg, diastolic blood pressure of ≥ 90 mmHg, and older age ≥ 50.

**Table 2:** Six-variable AKI Risk Score (AKI-RiSc) breakdown.

Predictor	Allocated Score					
	0	1	2	3	5	7
Creatinine (μmol/L)	< 100		100–149		150–249	≥ 250
Bicarbonate (mmol/L)	≥ 20	< 20				
Pulse (beats/min)	< 100		100–119	≥ 120		
SBP (mmHg)	< 150	≥ 150				
DBP (mmHg)	< 90	≥ 90				
Age (years)	< 50		≥ 50			

*SBP* systolic blood pressure, *DBP* diastolic blood pressure.

In terms of performance, AKI-RiSc performed reasonably well at an AUC of 0.730 (95% CI 0.714–0.747) when evaluated on the test cohort. At a cut-off of 4 points, it has a sensitivity of 82.6%, specificity of 46.7%, positive predictive value of 10.9%, and negative predictive value of 97.1%. When the APS^25^, a similar point-based AKI risk score, was evaluated on the same test cohort as a basis of comparison, it scored an AUC of 0.665 (95% CI 0.646–0.679). The APS consisted of age, respiratory rate, mental status (AVPU), chronic kidney disease (CKD), congestive cardiac failure (CCF), diabetes mellitus (DM), and liver disease. Table 3 presents the score cut-offs of AKI-RiSc and their respective sensitivity, specificity, positive predictive value, and negative predictive value. Figure S1 shows the correlation between the calculated AKI-RiSc value and the proportion of patients who developed AKI within 7 days. Almost 30% of patients with an AKI-RiSc of 9 developed AKI.

**Table 3:** Score cut-offs of the predicted risk of AKI based on AKI-RiSc, including percentage of patient within score threshold, sensitivity, specificity, positive predictive value, and negative predictive value.

Predicted risk of AKI (%)	Score cut-off	Percent of patients (%)	Sensitivity (95% CI)	Specificity (95% CI)	Positive predictive value (95% CI)	Negative predictive value (95% CI)
≥ 2.5	≥ 2	93	98.5% (97.8–99.2%)	7.0% (6.6–7.4%)	7.7% (7.7–7.8%)	98.4% (97.5–99.1%)
≥ 5	≥ 4	55	82.6% (80.1–84.9%)	46.7% (45.8–47.6%)	10.9% (10.6–11.3%)	97.1% (96.7–97.5%)
≥ 7.5	≥ 6	26	57.6% (54.7–60.6%)	77.0% (76.3–77.6%)	16.5% (15.7–17.4%)	95.8% (95.5–96.1%)
≥ 10	≥ 7	20	52.0% (48.9–55.0%)	82.2% (81.5–82.8%)	18.8% (17.7–19.8%)	95.6% (95.3–95.8%)
≥ 15	≥ 8	14	41.1% (38.3–44.2%)	88.2% (87.6–88.7%)	21.6% (20.1–23.1%)	95.0% (94.7–95.2%)
≥ 30	≥ 11	2	8.8% (7.2–10.7%)	98.1% (97.9–98.4%)	27.4% (22.9–32.1%)	93.1% (93.0–93.3%)

*AKI* acute kidney injury.

## Discussion

In this study, we used a large retrospective dataset and the AutoScore framework to construct a point-based clinical score, AKI-RiSc, to assess the 7 day risk of AKI development in a general patient population after admission from the ED. AKI-RiSc performed significantly better than the APS score when evaluated on the same test cohort.

The development of AKI-RiSc using AutoScore and our dataset brings several advantages. First, our sample size of 119,468 is considerably large which improves the reliability of the results obtained from our study. Second, the AutoScore scoring framework is built upon machine learning, which achieves a better parsimonious solution for clinical prediction tasks with large data sets^27^. Third, the selection of variables for AKI-RiSc was also performed in a transparent manner by identifying the most parsimonious solution from the parsimony plot (Fig. 2), allowing clinicians to better understand the processes involved in generating the score.

AKI-RiSc was designed for the general patient population in the form of a simple point based score that can be easily applied and interpreted by clinicians. Only the 6 most important variables were chosen for the final model, as additional variables only contributed marginal increase in model performance with the downside of increasing model complexity (Fig. 2). These variables, (SCr, bicarbonate, pulse, systolic blood pressure, diastolic blood pressure, and age) consisted of common biochemical results as well as patient parameters that could be obtained directly at the bedside within 2 h of admission at the ED. The score’s simplicity makes it practical in the clinical setting in the form of an automated score integrated in a hospital’s EHR system, or just via manual calculation by the clinician^28^.

When compared to the APS developed by Forni et al. in 2013, which was also a similar point-based AKI risk score for general patients in the ED^25^, AKI-RiSc outperformed APS by a significant margin in terms of AUC performance (0.730 vs 0.665), approximately 10% increase. One key difference between the two scores was the use of biochemical variables in AKI-RiSc—SCr and serum bicarbonate. SCr is a well-accepted marker of kidney function and has also been the most crucial variable in other AKI prediction models^10,29^. Low bicarbonate, which typically indicates metabolic acidosis, was also found independently associated with increased AKI risk in other studies^30^. Including these biochemical results could have potentially improved the performance of AKI-RiSc. It is worth noting that the APS achieved an AUC of 0.71 when evaluated on its own UK cohort^31^. One explanation of the poor performance of the APS in Singapore cohort could be the inherent differences between the population demographics it was derived and tested on^32^. For example, the average population age of the UK cohort was overall older than that of the Singapore cohort.

Comparing to other ML models, however, the performance of AKI-RiSc lags. Tomašev et al. developed a recurrent neural network AKI prediction model that calculated and updated the risk of AKI every 48 h, performing well with an AUROC of 0.921^29^. Despite these remarkable results, the translation of ML models to practical clinical medicine has been limited. ML models often require significant amount of information to function optimally, which may be difficult to obtain consistently^33^. Furthermore, the complexity of the models’ algorithms make it challenging for implementation in many hospital’s IT infrastructure^34^. Many of these ML models are also developed using black box approaches as opposed to more traditional logistic regression models which clinicians are familiar with^34^. This can potentially limit data interpretation and the willingness of clinicians to adopt the model’s predicted risk^13^. Therefore, while AKI-RiSc may not perform as well as pure ML models in AKI prediction, it has the benefit of being much more easily implemented in the clinical setting with a reasonable predictive performance.

Several actions can be taken after a high-risk patient has been identified using AKI-RiSc in the ED. First, interventional bundle care plans can be instituted for patients identified with a high AKI-RiSc in the form of a checklist to guide clinicians in the treatment of AKI^7^ and potentially improve patient outcomes^35^. Second, a high AKI-RiSc score can prompt the emergency physician to make earlier nephrology consults, which can improve the outcomes of patients with renal impairment^36^. Third, the utility of the AKI-RiSc can be further enhanced when paired with novel AKI biomarkers, in which high risk patients who may benefit from AKI biomarker test can be initially identified using AKI-RiSc.

There are limitations in this study. As with most other AKI studies, AKI was defined only using serum SCr as urine output information was unavailable. Furthermore, more than half of the admissions were first-time admissions, meaning that they did not have a median annual baseline SCr. A significant number of admissions relied on the ED SCr reading as the baseline. In reality, admission SCr could already have been pathologically raised due to prior kidney insults preceding the admission. Hence, using the ED SCr as a baseline reference meant that the actual incidence of AKI could be underestimated^37^. While we could accurately identify the diagnosis of AKI in patients by trending changes in SCr, we did not have any information on the aetiology of the AKI. However, AKI-RiSc was designed to be an alert system designed to recommend treatment, not as a diagnostic tool^25^. Finally, owing to a lack of data, we were unable to construct scores specifically for community-acquired AKI and hospital-acquired AKI. This study therefore sought to develop and validate a single score that could be easily calculated for quick risk stratification in the ED. A future study might focus on developing more accurate scoring tools tailored to different causes of AKI where additional important variables can be incorporated.

The next step would be to prospectively validate the AKI-RiSc in various hospitals in Singapore. AKI-RiSc can then be sequentially applied to hospitals in regional Southeast Asian countries, and eventually to the international setting. We believe that the simplicity of AKI-RiSc and use of common clinical variables makes it a good candidate for validation in hospitals across different settings. Apart from assessing its predictive performance in a prospective validation cohort, it would also be essential to gain feedback from physicians on the score’s perceived utility and ease of use in helping them better care for their patients. AKI bundles can also be developed to complement the AKI-RiSc to assess its effectiveness in improving patient outcomes.

## Conclusions

In conclusion, we developed a simple and interpretable point-based AKI risk score which can be easily implemented on the ground for early identification of high-risk patients in the ED. It also has the potential be applied in healthcare settings internationally.

## Supplementary Information

Supplementary Information.
