# Supplementary material for: Development and validation of an interpretable clinical score for early identification of acute kidney injury at the emergency department
Source: Sci Rep. 2022 May 2;12:7111. doi: 10.1038/s41598-022-11129-4 (PMC9061747; doi:10.1038/s41598-022-11129-4)
Supplement: Supplementary file 1 — Supplementary Information. [file 41598_2022_11129_MOESM1_ESM.pdf]

Supplementary Materials

Table S1: Univariable analysis of candidate variables

Figure S1: Proportion of patients developing AKI within 7 days of admission and their respective AKI-RiSc value

Figure S2: The AutoScore workflow

Table S1: Univariable analysis of candidate variables

Variable	Odds Ratio (95% Confidence interval)	P-value
Gender, Male	1.00 (0.96-1.04)	0.98
Age, years (Ref: <32)		
32 – 52	2.38 (2.00-2.84)	<0.001
53 – 80	4.10 (3.48-4.83)	<0.001
81 – 88	4.66 (3.94-5.52)	<0.001
>88	4.69 (3.92-5.61)	<0.001
Pulse, beats per min (Ref: 59 – 68)		
31 – 58	1.15 (1.03-1.29)	0.01
69 – 98	1.18 (1.11-1.26)	<0.001
99 – 115	1.59 (1.48-1.71)	<0.001
>115	2.35 (2.15-2.57)	<0.001
Respiration, breaths per min (Ref: 16)		
<16	2.45 (2.14-2.8)	<0.001
17	1.04 (0.96-1.12)	0.35
18 – 19	1.15 (1.08-1.22)	<0.001
>19	2.23 (2.08-2.39)	<0.001
SpO2, % (Ref: >96)		
95 – 96	1.19 (1.13-1.25)	<0.001
<95	2.14 (1.92-2.38)	<0.001
SBP, mmHg (Ref: 98 – 110)		
<98	1.39 (1.25-1.53)	<0.001
111 – 150	0.96 (0.90-1.02)	0.16
151 – 176	1.28 (1.2-1.38)	<0.001
>177	1.8 0(1.64-1.96)	<0.001
DBP, mmHg (Ref: 59 – 79)		
<50	1.79 (1.64-1.95)	<0.001
50 – 58	1.14 (1.08-1.21)	<0.001
80 – 92	1.16 (1.09-1.22)	<0.001
93 – 148	1.55 (1.43-1.68)	<0.001
Bicarbonate, mmol/L (Ref: 25.4 – 28.4)		
<15.8	3.11 (2.85-3.4)	<0.001
15.8 – 19.2	1.95 (1.81-2.1)	<0.001
19.3 – 25.3	1.18 (1.10-1.26)	<0.001
>28.4	1.27 (1.14-1.41)	<0.001
Creatinine, micromol/L (Ref: 46 – 61)		
<46	2.25 (1.97-2.56)	<0.001
62 – 143	2.04 (1.86-2.22)	<0.001
144 – 254	6.11 (5.57-6.70)	<0.001
255 – 366	14.04 (12.71-15.51)	<0.001
Potassium (Ref: 3.5 – 4.6)		

<3	1.26 (1.14-1.39)	<0.001
3 – 3.4	0.96 (0.90-1.03)	0.27
4.7 – 5.5	1.46 (1.38-1.53)	<0.001
>5.5	1.58 (1.46-1.71)	<0.001
Sodium (Ref: 129-137)		
<122	1.18 (1.07-1.29)	<0.001
122 – 128	0.98 (0.92-1.05)	0.62
138 – 140	1.03 (0.98-1.09)	0.26
>140	1.38 (1.28-1.48)	<0.001
Renal Disease	4.07 (3.91-4.24)	<0.001
Myocardial Infarction	3.36 (3.17-3.55)	<0.001
Congestive Heart Failure	2.82 (2.70-2.95)	<0.001
Peripheral Vascular Disease	2.49 (2.34-2.66)	<0.001
Stroke	1.48 (1.40-1.55)	<0.001
Dementia	1.17 (1.05-1.29)	<0.001
Pulmonary Disease	1.39 (1.31-1.47)	<0.001
Rheumatic Disease	1.43 (1.24-1.66)	<0.001
Peptic Ulcer Disease	1.65 (1.52-1.79)	<0.001
Mild Liver Disease	1.35 (1.25-1.47)	<0.001
Severe Liver Disease	1.69 (1.51-1.89)	<0.001
Diabetes Mellitus	1.17 (1.12-1.24)	<0.001
Diabetic Complications	1.81 (1.74-1.89)	<0.001
Paralysis	1.41 (1.31-1.52)	<0.001
Cancer	1.16 (1.09-1.23)	<0.001
Metastatic Disease	1.13 (1.06-1.21)	<0.001
High dependency admissions in last year (Ref: 0)		
1	1.64 (1.47-1.82)	<0.001
2	1.76 (1.52-2.03)	<0.001
>2	2.12 (1.79-2.52)	<0.001
ICU admissions in last year (Ref: 0)		
1	2.11 (1.74-2.55)	<0.001
2	2.50 (1.96-3.17)	<0.001
>2	2.15 (1.56-2.96)	<0.001
Hospital admissions in last year (Ref: 0)		
1	1.49 (1.42-1.57)	<0.001
2	1.77 (1.65-1.89)	<0.001
>2	2.02 (1.90-2.14)	<0.001
Number of surgeries in last year (Ref: 0)		
1	1.44 (1.34-1.55)	<0.001
2	1.75 (1.56-1.96)	<0.001
>2	2.58 (2.28-2.91)	<0.001
Intubation	4.07 (2.28-7.28)	<0.001
Resuscitation	2.11 (1.93-2.31)	<0.001

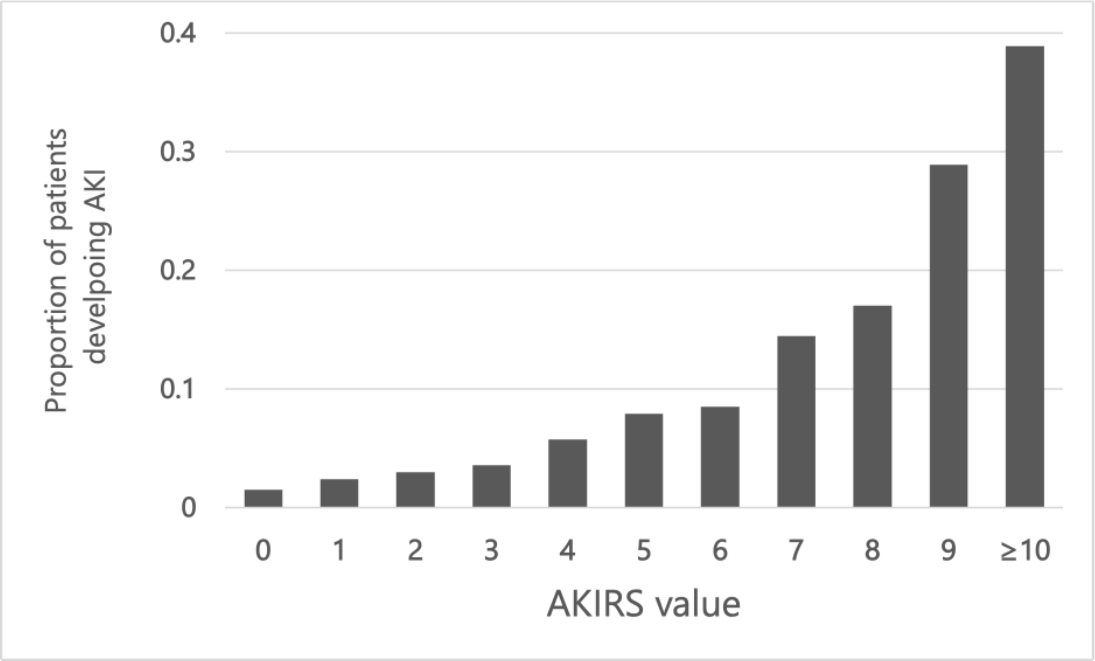

Figure S1: Proportion of patients developing AKI within 7 days of admission and respective AKI-RiSc value

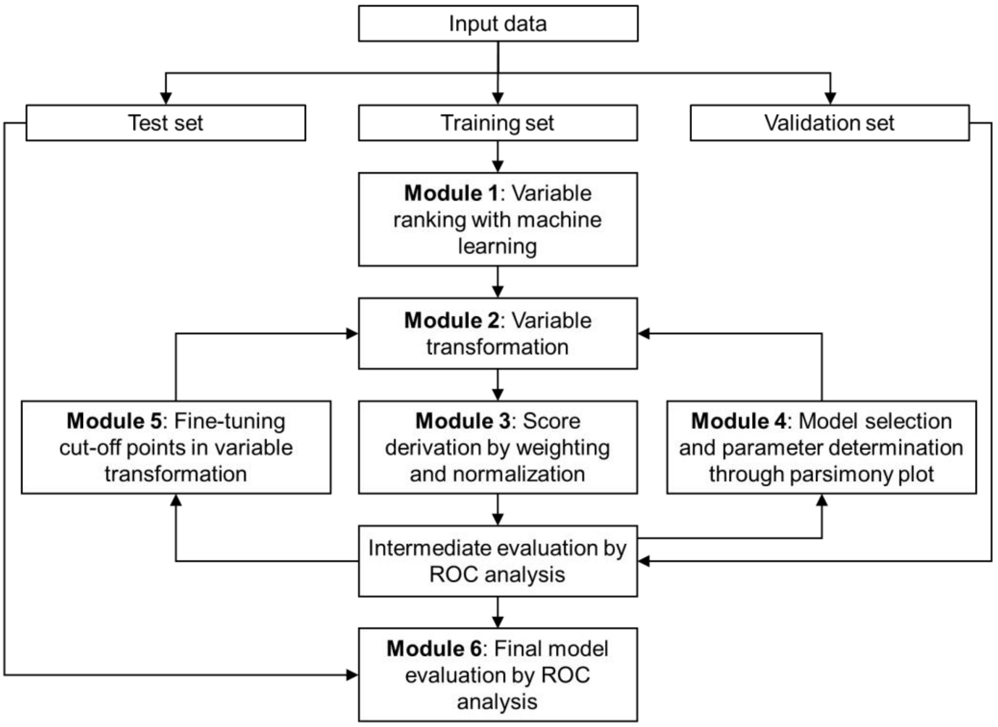

Figure originally published in JMIR Medical Informatics under CC-BY license:

- Xie F, Chakraborty B, Ong MEH, Goldstein BA, Liu N. AutoScore: A Machine Learning–Based Automatic Clinical Score Generator and Its Application to Mortality Prediction Using Electronic Health Records. JMIR Med Inform 2020;8(10):e21798.

With AutoScore, users could seamlessly generate sparse and parsimonious risk scores, which could be easily implemented in clinical practice. The six modules in AutoScore enable users to develop and validate clinical scores based on the following five steps:

- Step(i): Rank variables with machine learning (Module 1)
- Step(ii): Select the best model with parsimony plot (Modules 2+3+4)
- Step(iii): Generate the initial score with the final list of variables (Rerun Modules 2+3)
- Step(iv): Fine-tune the score with domain knowledge (Module 5)
- Step(v): Evaluate the final score with ROC analysis (Module 6)

The following links provide additional information on AutoScore:

- Research paper (https://medinform.jmir.org/2020/10/e21798/)
- R package (https://github.com/nliulab/AutoScore)

Figure S2: The AutoScore workflow
